# Fluorescence based Aptasensors for the determination of hepatitis B virus e antigen

**DOI:** 10.1038/srep31103

**Published:** 2016-08-08

**Authors:** Rongrong Huang, Zhijiang Xi, Yan Deng, Nongyue He

**Affiliations:** 1 State Key Laboratory of Bioelectronics, School of Biological Science and Medical Engineering, Southeast University, Nanjing 210096, China; 2 Medical School of Yangtze University, Jingzhou 434023, China; 3 Economical Forest Cultivation and Utilization of 2011 Collaborative Innovation Center in Hunan Province, Hunan Key Laboratory of Green Packaging and Biological Nanotechnology, Hunan University of Technology, Zhuzhou 412007, P. R. China

## Abstract

This research is aimed at selecting specific aptamer of hepatitis B e antigen by SELEX and its applications. Hepatitis B e antigen (HBeAg) seroconversion is used as an indicator of virological response when treating patients suffering from chronic hepatitis B. HBeAg also indicates a high viremia and high infectivity in untreated patients. With HBeAg modified magnetic beads as targets, three groups of aptamers are successfully selected. These are the first reported DNA aptamers that can specifically bind to HBeAg. Based on the property that the conformation changes upon binding to its target, aptamer has emerged as ideal candidate in a variety of sensing applications. In this study, we present a simple strategy for aptamer-based fluorescence biosensors for the quantitative detection of HBeAg, in which a fluorescence labeled HBeAg aptamer serves as the molecular recognition element and a short DNA molecule that is complementary to the aptamer serves as the competitor. The LOD for HBeAg is 609 ng/mL. Later, the fluorescence system is deployed in HBeAg positive and negative blood serum (p < 0.05). The total detection assay could be completed in 2 min. These newly isolated aptamers could assist the diagnosis of chronic hepatitis B.

Hepatitis B is an infectious disease caused by the hepatitis B virus (HBV) that affects the liver. Recent estimates suggested that HBV infection caused 686 000 deaths in 2013^1^,^2^. As 25% of people who acquire HBV as children will develop primary liver cancer or cirrhosis as adults, chronic hepatitis B virus infection (CHB) is a major public health issue worldwide^3^. However, current treatment options for CHB are ineffective^4^. Both pegylated interferon therapy and nucleos(t)ide analogues could not achieve the goal to cure CHB.

HBeAg is a viral protein of clinical importance, which is soluble and closely related to non-secretory capsid antigen hepatitis B core antigen (HBcAg). The sequences of those two proteins are largely overlapping. The different immunoreactivity between HBeAg and HBcAg is created by a different conformation of the B cell epitopes. HBeAg may also induce a specific non-responsiveness of helper T cells. HBeAg appears in the serum during the high replicative phase of HBV infection, which makes it a serum marker of active viral replication. It is also used to identify highly infectious mothers. Approximately 90% of infants of hepatitis B surface antigen (HBsAg) carrier mothers with positive HBeAg will become carriers if no immunoprophylaxis is given. Furthermore, HBeAg is accompanied by serum levels of HBV DNA that are 100,000 to 1 million IU per milliliter or higher^5^.

CHB can be broadly divided into two major forms: hepatitis B e antigen (HBeAg)-positive and HBeAg-negative. HBeAg-positive CHB is accompanied by high-level HBV replication, and spontaneous seroconversion from HBeAg-positive to antibody-positive infection coincides with a reduction in HBV replication and clinical improvement^5^. Patients with HBeAg-negative CHB harbor a naturally occurring mutant form of HBV that does not produce HBeAg because of a mutation in the precore or core promoter region of the HBV genome. These patients generally require prolonged treatment in contrast to those with HBeAg-positive CHB.

Aptamers are single-stranded nucleic acid ligands that are generated by an *in vitro* selection process called SELEX (systematic evolution of ligands by exponential enrichment)^6^,^7^. Aptamers can be considered as nucleic acid analogues of antibodies. They possess high affinities to target molecules, with dissociation constants in the nanomolar or picomolar range^8^. Currently, aptamers have been selected for a wide range of targets, including peptides, nucleotides, amino acids, antibiotics, proteins, vitamins, low-molecular weight organic or inorganic compounds, and even whole cells. Thus, aptamers are capable of becoming excellent chemical biosensors and may play an increasingly important role in public health, environmental monitoring, and food security^9^,^10^.

Several aptamers related to HBV have been previously selected. For example, Liu and colleagues reported the selection of RNA aptamers that can specifically bind to the HBsAg protein and HBsAg-positive hepatocytes^11^. Zhang and colleagues screened five different aptamers against HBcAg from a random ssDNA library, one of which could inhibit the assembly of the nucleocapsid and reduce extracellular HBV DNA, indicating its potential efficacy in suppressing HBV replication^12^. However, HBeAg-specific aptamers have not been reported.

Gold nanoparticles (AuNPs) possess strong distance-dependent optical properties and are considered important colorimetric materials^13^. AuNPs have been incorporated into novel biosensor strategies because of their optical properties, ease of use, and simple synthesis^14^. For example, Wang and colleagues reported a simple, sensitive, and inexpensive multiplex pathogen detection scheme based on novel gold nanorod bioprobes^15^. Sensors based on aptamer-linked AuNPs have also been demonstrated for detecting duplex DNA formation, protein-ligand interactions, and metal ion-ligand complexation^16^,^17^,^18^.

We also developed a rapid and sensitive method for the detection of HBeAg using aptamer-based fluorescence assay. Fluorescence molecules are attractive as a signaling moiety. Methods that employ fluorescent reporters have proven to be particularly useful in generation of aptamer-based biosensors. For example, fluorescence labeled aptamers have been reported for sensing a large amount of targets, such as thrombin^19^, mercury ions^20^, and adenosine^21^, etc.

The most typical detection method for HBeAg is ELISA (enzyme-linked immunosorbent assay). However, there are several disadvantages of ELISA, such as time-consuming and false positive/false negative deviation^22^. We selected novel aptamers that recognize and bind to HBeAg with appreciable affinity and selectivity. The most promising of the selected aptamers was incorporated with AuNPs and fluorescence, respectively, enabling the selective detection of HBeAg over other proteins.

## Results

### Test of binding affinity

ssDNA pools from each round were tested for their binding affinities to HBeAg (see Supplementary Fig. S1). The binding affinities of aptamers increased during later rounds of SELEX compared with those of the starting pool. However, binding affinities increased only slowly after nine rounds, indicating that affinity was nearing its maximum. Therefore, the SELEX process was terminated after the eleventh round.

### Sequences and secondary structure of the selected aptamers

After eleven rounds, the selection process was completed, and the products were amplified, purified, and cloned. Twenty randomly picked clones were sequenced (Table 1). The results were divided into three groups. As can see in Fig. S2, each group of aptamers exhibited a stronger affinity for HBeAg immobilized on magnetic beads than that of aptamers from the starting pool. The affinity of aptamers from group 2 was found to be the strongest.

**Figure 1:**
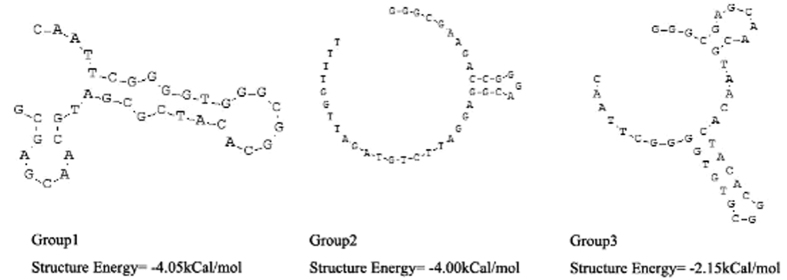
Predicted secondary structures of the core region of selected aptamers from groups 1, 2, and 3.

Secondary structures of the selected aptamers were analyzed by DNAMAN software (Fig. 1). Aptamers from group 2 shared the same secondary structure.

### Detection of HBeAg using AuNP-based colorimetric aptasensor

As shown in Fig. S3, the 14-nm AuNPs used in our study are spherical and evenly distributed. Naked AuNPs aggregated to form bigger clusters in the presence of high concentration of NaCl. However, ssDNA-linked AuNPs did not aggregate. When 0.09 mM NaCl was added, the overall color of the solution changed from purple to red as the concentration of ssDNA increased (see Supplementary Fig. S4). Furthermore, when 4 *μ*M ssDNA were added, the color of the solution was the same as that of the naked AuNPs when NaCl was not added. Therefore, we determined that the optimal concentration of added ssDNA was 4 *μ*M.

**Figure 2:**
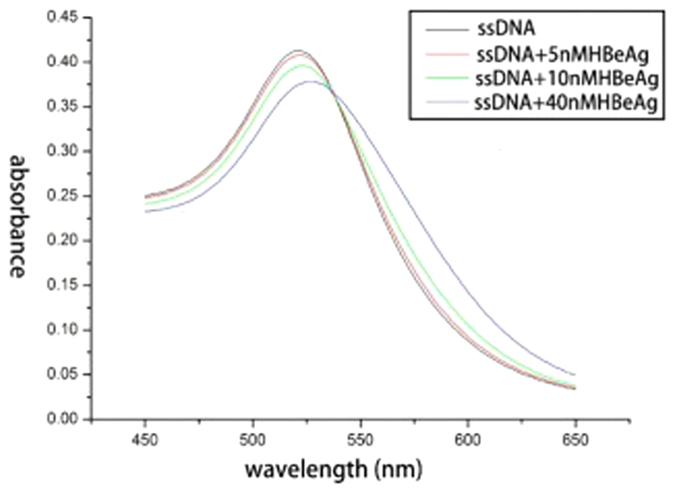
Detection of HBeAg by using an AuNP-based colorimetric aptasensor.

**Figure 3:**
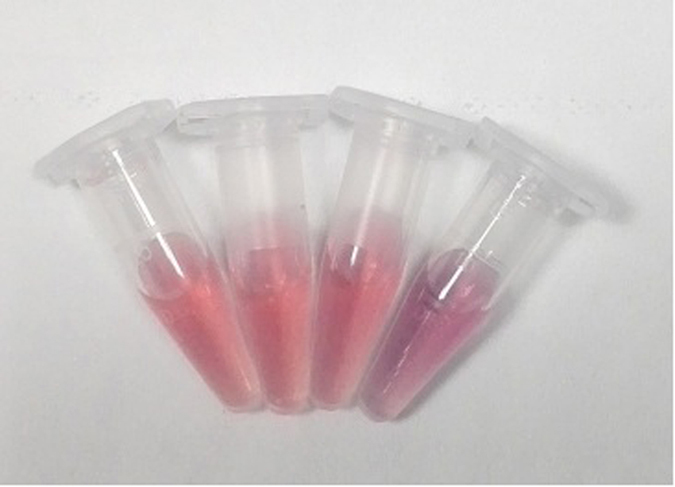
Photograph of color changes caused by adding HBeAg to aptamer-linked AuNPs. From left to right: aptamer-linked AuNPs + 0.09 M NaCl; aptamer-linked AuNPs + 50 nM parenzyme + 0.09 M NaCl; aptamer-linked AuNPs + 50 nM HBcAg + 0.09 M NaCl; aptamer-linked AuNPs + 50 nM HBeAg + 0.09 M NaCl.

The AuNP-based colorimetric aptasensor exhibited a maximum absorption peak at about 522 nm, as shown in Fig. 2. This indicates that the size of AuNP did not change after being coated with ssDNAs. Upon addition of HBeAg, the AuNPs combined with ssDNA to form a more stable aptamer-protein complex. This caused the release of ssDNA from the AuNPs. From these results, it could be concluded that aptamer-linked AuNPs aggregate to form bigger clusters in the presence of 0.09 mM NaCl and exhibit a new absorption peak with a maximum at higher wavelengths, whereas further increase in HBeAg concentration causes absorbance at 522 nm to decrease considerably. Importantly, this shift in the new peak becomes more obvious as AuNPs continue to aggregate into larger particles. Thus, in the presence of HBeAg, the aptasensor aggregated to form bigger clusters, and the overall solution color changed from red to dark purple. It should be noted that the addition of other proteins, such as trypsin and HBcAg did not cause the change in the color of the solution, indicating the high specificity of aptasensor for HBeAg (Fig. 3).

### Detection of HBeAg using fluorescence labeled aptamer

In this work, Dabcyl modified probe Q was used as fluorescence quencher. In the absence of HBeAg, a fluoresent dye (FAM) labeled HBeAg aptamer (named probe F) hybridized with probe Q to form a DNA duplex, leading to quenching. Once HBeAg was introduced, the FAM-aptamer was combind with HBeAg, which resulted in the FAM-aptamer being separated from probe Q and restoration of fluorescence. As shown in Table S1, the fluorescence intensity of aptamers decreased as the probe Q concentration was increased and eventually reached a saturation point after which any further increase in the probe Q concentration caused little decrease in the fluorescence intensity. Therefore, we determined the optimal concentration proportion of probe F and probe Q was 1: 3.

**Figure 4:**
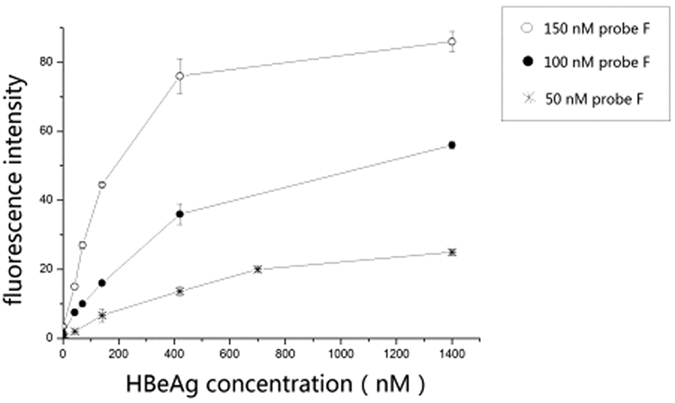
Plots of fluorescence intensity change as a function of HBeAg concentrations. The concentration of probe F was 50, 100 and 150 nM, respectively.

**Figure 5:**
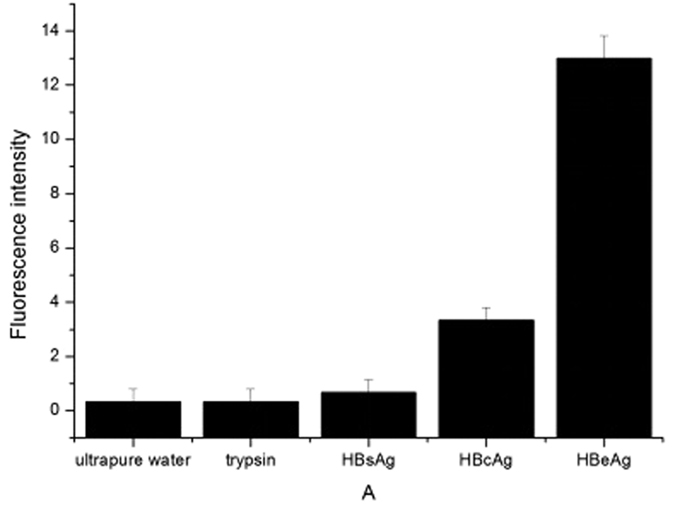
Fluorescence intensity changes caused by adding different proteins to the mixture of probe F and probe Q. The concentration of proteins was 420 nM.

**Figure 6:**
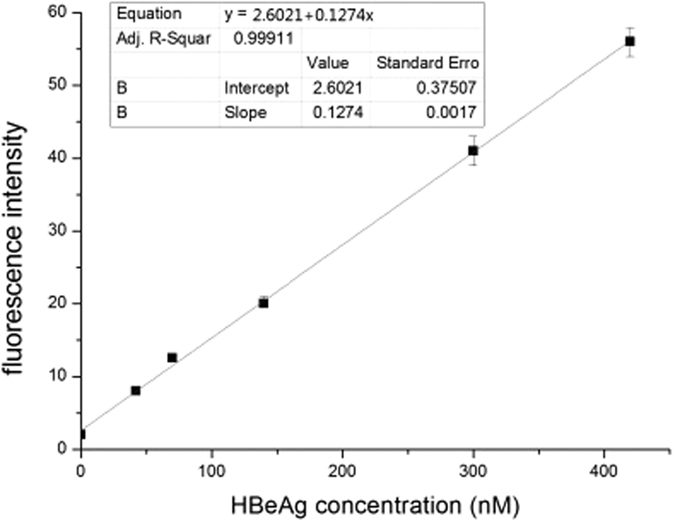
The linear relationship between the fluorescence intensity change and the HBeAg concentration.

When HBeAg was added to the mixture of probe F and probe Q, the restoration of fluorescence was observed. As shown in Fig. 4, under the condition of 100 nM probe F, the linear relationship between the fluorescence intensity and the concentration of HBeAg was the best. Therefore, we determined the optimal concentration of probe F was 100 nM. To test the possibility of the fluorescence signal of the probe F for the quantitative detection of HBeAg, sufficient replicates were carried out. However, the addition of other proteins, such as trypsin, HBcAg and HBsAg did not cause the change in the fluorescence intensity, indicating the high specificity of the aptamer-based fluorescence biosensor for HBeAg (Fig. 5). Figure 6 showed that the fluorescence intensity increased as the concentration of HBeAg increased. The fluorescence signal had a good linear relationship with HBeAg concentration in the range of 42~420 nM, and the regression equation for this relationship was y = 2.6021 + 0.1274x (R = 0.9991). The limit of detection for HBeAg was as low as 26.5 nM.

### Detection of HBeAg in blood serum using fluorescence labeled aptamer

**Figure 7:**
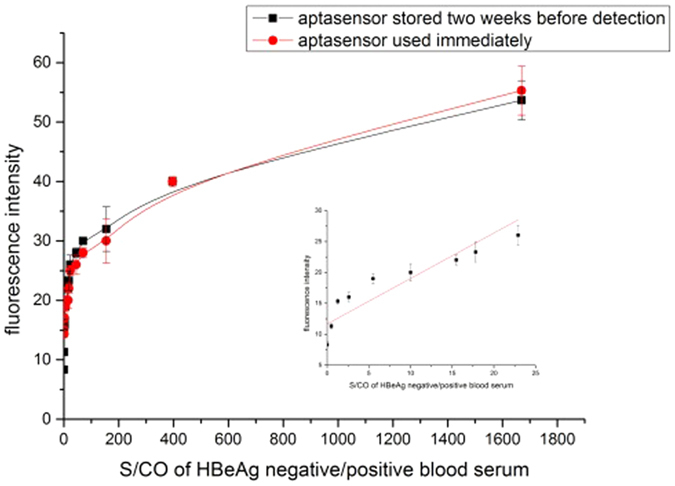
Fluorescence intensity changes caused by adding different HBeAg negative/positive blood serum samples to the mixture of probe F and probe Q. The mixture of probe F and probe Q was stored at 4 °C for two weeks or used immediately for detection. After gently shaking for 2 min, the fluorescence intensity was measured.

The fluorescence system was stored at 4 °C for two weeks and then deployed in diluted blood serum. Figure 7 showed that the fluorescence intensity increased sharply as the S/CO of HBeAg positive blood serum increased, and when the S/CO of blood serum reached 22.92, the rise trend began to slow down. As can been seen from the result, the fluorescence aptasensor was stable in two weeks. This proved the presented assay approach was simple and fast.

## Discussion

We selected HBeAg-specific DNA aptamers using magnetic beads as an immobilization matrix for target molecules. Magnetic beads can provide a homogeneous surface with different functional groups for the immobilization of selected target molecules^23^,^24^. Additionally, unbound ssDNA was easily separated from bead-bound ssDNA through magnetic separation. This method offers the potential for parallel processing of multiple targets without the need for expensive robotics.

In this study, we have generated new HBeAg-specific aptamers and chosen the most promising aptamer for further development and application. Furthermore, an AuNP-based colorimetric aptasensor has been developed for colorimetric sensing of HBeAg. This method was based on HBeAg-aptamer interaction and distance-dependent optical properties of AuNPs. In the presence of HBeAg, the aptasensor aggregated to form bigger clusters, and the overall solution color changed from red to dark purple. Addition of other proteins did not cause the solution to change color, indicating that this aptasensor is highly specific for HBeAg. We also developed a rapid aptamer-based fluorescence biosensor to convert the aptamer-target recognition event into an fluorescence signal. In the presence of HBeAg, fluorescence recovery was observed. By taking advantage of affinity and specificity of aptamer, the biosensor showed excellent selectivity toward target. The biosensor also showed sensitivity and specificity to HBeAg in patient blood serum. As the fluorescence system was stable in two weeks or even more, the detection time could be shortened to 2 min.

Selection and application of HBeAg-specific aptamers described in this study is expected to promote the expoitation of aptamer-based detection for other proteins like HBsAg, EGFR (epithelial growth factor receptor) and so on. They can be designed as targeting ligands to differentiate diseased cells from healthy cells, thus enabling the selective delivery of therapeutic compounds to target cells^25^, such as aptamer-based DNA dendritic nanostructure as a multifunctional vehicle for targeted cancer cell imaging and drug delivery^26^. The emerging integration of nanotechnology and chemical biology is envisioned to produce further new clinical applications in the near future^27^.

## Methods

### Ethics statement

All experiments were carried out in accordance with relevant guidelines and regulations. The study was approved by the Research Ethics Committee of Southeast University, China. Because the tested samples were de-identified and would normally have been discarded. Informed consent of the study subjects was not required due to the nature of the samples.

### Reagents and materials

The starting random ssDNA library and PCR amplification primers were synthesized by Shanghai Sangon Biotech Co. Ltd. (Shanghai, China). The ssDNA library contained a 40-base central random sequence flanked by primer sites on either side. The sequences of these designed oligonucleotides were as follows:

ssDNA library: 5′-GGGAATTCGAGCTCGGTACC-(40N)-CTGCAGGCATGCAAGCTTGG-3′.

Primer 1: 5′-GGGAATTCGAGCTCGGTACC-3′.

Primer 2: 5′-CCAAGCTTGCATGCCTGCAG-3′.

Primer 3: 5′-biotin-GGGAATTCGAGCTCGGTACC-3′.

Two DNA probe sequences were synthesized by Shanghai Sangon Biotech Co. Ltd. (Shanghai, China). The sequences were as follows:

Probe F: 5′-FAM-GGGGGGGCGAAGACCGGGACGGGAGGATTCTGTAGATTGGTTTT-3′. Probe Q: 5′-CTTCGCCCCCCC-Dabcyl-3′.

Bovine serum albumin (BSA), HEPES (free acid), 1-(3-dimethylaminopropyl)-3-ethylcarbodiimide hydrochloride (EDC), and horseradish peroxidase (HRP)-conjugated streptavidin were purchased from Shanghai Sangon Biotech Co. Ltd. (Shanghai, China). Carboxylated magnetic beads were prepared by our laboratory. 2-*N*-morpholino ethanesulfonic acid (MES) and *N*-hydroxysuccinimide (NHS) were purchased from Life Science Products & Services. Recombinant HBeAg, HBsAg and HBcAg were purchased from Yikang Biological Technology Company (Zhengzhou, China). HBeAg positive and negative blood sera were kindly offered Gulou hospital (Nanjing, China).

HBeAg-coated magnetic beads were made by our laboratory.

Quick PCR Purification Kit was purchased from GenScript (Nanjing, China).

### SELEX procedure and binding affinity assays

Specific procedures refer to previous article^2^,^3^ ssDNA pools with different concentrations (500 pmol for initial round; 100 pmol for 2nd–6th rounds; 50 pmol for 7th–11th rounds) were used for each selection step.

### Preparation of AuNPs

A two-neck flask, magnetic stir bar, stopper, and condenser were soaked overnight in aqua regia. The glassware was rinsed with copious amounts of ddH_2_O twice. Five hundred microliters of 1% HAuCl_4_ solution were added into 49.5 ml of ddH_2_O so that the final HAuCl_4_ concentration was 0.01%. One neck of the flask was connected with the condenser and the other with the stopper. The flask was placed on a hot plate to reflux while stirring. When the solution began to boil, 1.5 mL of 1% sodium citrate was quickly added. The color changed from pale yellow to dark purple and then to deep red in 1 min. The system was allowed to reflux for another 20 min and then return to room temperature (25 °C) for 2 h. The prepared AuNPs were stored at 4 °C.

### Detection of HBeAg using AuNPs-based colorimetric aptasensor

40 *μ*L of the HBeAg-specific aptamer were added into 2.5 mL of AuNPs and incubated at room temperature for 20 min. The solution was divided into 4 groups. 1 *μ*L each of ultrapure water, trypsin, HBcAg and HBeAg was added to 400 *μ*L of nanoparticles in each group, respectively. After standing for 30 min, 0.09 mM NaCl was added. The absorbance of nanoparticles was measured by UV-visible spectroscopy (Shimadzu UV-1800).

### Detection of HBeAg using fluorescence labeled aptamer

To determine the optimal concentration proportion of probe F and probe Q, two microliters each of ultrapure water and 5, 10, 15, 20 *μ*M probe Q were added to a series of Eppendorf centrifuge tubes containing 200 *μ*L of buffer A (50 mM NaCl, 7 mM MgCl_2_, 50 mM Tris-HCl) and 50 *n*M of probe F, respectively. After incubated at room temperature for 20 min, the fluorescence intensity was determined using a Synergy HT Multi-Mode Microplate Reader, with an excitation wavelength of 485 nm and an emission wavelength of 528 nm.

To demonstrate the detection capability of the fluorescence labeled aptamer, 1.2 *μ*L of probe F (50 *μ*M) was mixed with 3.6 *μ*L of probe Q (50 *μ*M) in 1.2 mL of buffer A. The solution was divided into 6 equal parts. Five microliters each of ultrapure water and different concentrations from 0.168 to 3.36 *μ*M of HBeAg were added to each equal part, respectively. After standing for 30 min, the fluorescence intensity was measured.

To demonstrate the specificity of the fluorescence labeled aptamer, 5 *μ*L of probe F (10 *μ*M) was mixed with 15 *μ*L of probe Q (10 *μ*M) in 1 mL of buffer A. The solution was divided into 5 groups. 5 *μ*L each of ultrapure water, 1.68 *μ*M trypsin, HBsAg (hepatitis B surface antigen), HBcAg (hepatitis B core antigen) and HBeAg were added to each equal part, respectively. After standing for 30 min, the fluorescence intensities were measured.

To determine the optimal concentration of probe F, five microliters each of 50, 100 and 150 *μ*M probe F were added to a series of Eppendorf centrifuge tubes containing 1 mL of buffer A. And after 20 min incubating with probe Q, the solution in each tube was divided into 5 partes aequales. Five microliters each of ultrapure water and different concentrations from 0.168 to 3.36 *μ*M of HBeAg were added to each equal part. After standing for 30 min, the fluorescence intensities were measured.

### Detection of HBeAg in blood serum using fluorescence labeled aptamer

460.8 *μ*L of probe F (10 *μ*M) was mixed with 1382.4 *μ*L of probe Q (10 *μ*M) in 7.2 mL of buffer A. The solution was then divided into 72 equal parts. 40 *μ*L each of HBeAg positive blood serum were added to each equal part, respectively. After gently shaking for 2 min, the fluorescence intensity was measured. To test the stability of the fluorescence based aptasensor, the mixture of probe F and probe Q was stored at 4 °C for two weeks and then used for detection.

## Additional Information

**How to cite this article**: Huang, R. *et al.* Fluorescence based Aptasensors for the determination of hepatitis B virus e antigen. *Sci. Rep.* **6**, 31103; doi: 10.1038/srep31103 (2016).

## Supplementary Material

Supplementary Information

## Figures and Tables

**Table 1:** The DNA sequences of twenty clones.

Group	Aptamer clone	Core region of the selected aptamers
1	Aptamer 1	5′-CCAATTCGGGGTGGGCGGCACATCGCGATGCAACGAGCG-3′
2	Aptamer 2-19	5′-GGGCGAAGACCGGGACGGGAGGATTCTGTAGATTGGTTTT-3′,
		5′-GGGCGAAGGCCGGGACGGGAGGATTCTGTAGATTGGTTTT-3′,
		5′-GGGCGAAGACCGGGATGGGAGGATTCTGTAGATTGGTTTT-3′,
		5′-GGGCGAAGACCGGGACGGAGGATTCTGTAGATTGGTTTT-3′
3	Aptamer 20	5′-GGGCGAGCAACGTAACACTACACGGCGTGTGGGGCTTAAC-3′
