# Supplementary material for: Fluorescence based Aptasensors for the determination of hepatitis B virus e antigen
Source: Sci Rep. 2016 Aug 8;6:31103. doi: 10.1038/srep31103 (PMC4976349; doi:10.1038/srep31103)

Supplementary Information

Fluorescence based Aptasensors for the determination of hepatitis B virus e antigen

Rongrong Huang^a,†^, Zhijiang Xi^a,b,†^, Yan Deng^a,c,*^, Nongyue He^a^,*

^a^ State Key Laboratory of Bioelectronics, School of Biological Science and Medical Engineering, Southeast University, Nanjing 210096, China

^b^ Medical School of Yangtze University, Jingzhou 434023, China

^c^ Economical Forest Cultivation and Utilization of 2011 Collaborative Innovation Center in Hunan Province, Hunan Key Laboratory of Green Packaging and Biological Nanotechnology, Hunan University of Technology, Zhuzhou 412007, P. R. China

*Corresponding author: hndengyan@126.com (Y. Deng); [nyhe1958@163.com](mailto:nyhe1958@163.com) (Y. He)

^†^These authors contributed equally to this work.

**Figure S1.** Influence of annealing temperature. (M) DNA marker; (1-6) PCR products annealed at different temperatures: 63, 64, 65, 66, 67, 68, and 70 ºC.


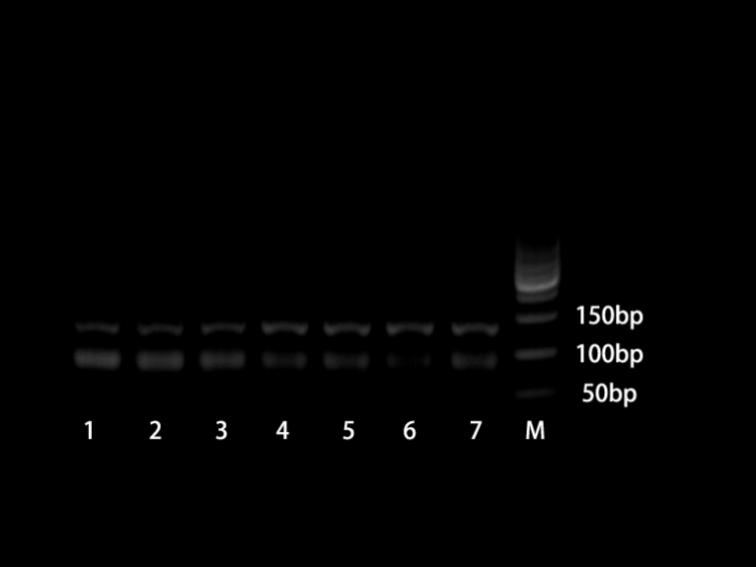


**Figure S2.** Influence of ssDNA template on specificity. (M) DNA marker; (1-4) 20, 15, 10, and 5 ng ssDNA template.


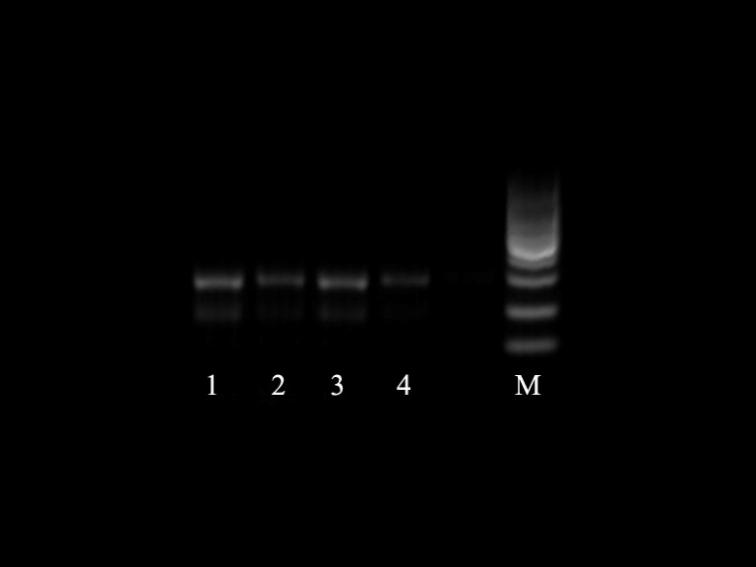

Supplement: Supplementary Information [file srep31103-s1.doc]
